# The Reference Hand-Book of the Medical Sciences

**Published:** 1902-03

**Authors:** 


THE REFERENCE HAND-BOOK OF THE MEDICAL SCIENCES. Embracing the Entire Range of Scientific and Practical Medicine and Allied Sciences. By various writers. A new edition, completely revised and rewritten. Edited by Albert H. Buck, M. D., New York City. Volume III. Illustrated by chromolithographs and six hundred and seventy-six half tone and wood engravings. Pages, 861, double column. New York: William Wood & Co. 1901.

This volume runs from *Chloralamid* to *Equinox Springs,* and when compared with the same volume of the old edition it shows much improvement and a detail of work that brings everything to the present time. There are one hundred and forty of the best known men in the country who contributed to this volume, and the evidence of revising and rewriting is seen throughout the work.

No student in the profession can fail to appreciate the wide range of the Reference Hand-Book. When once he has taken it up and become familiar with the arrangement of the subject matter, he promptly becomes a friend of the work. The common subjects are treated of, as well as the more uncommon. But it is in the latter that the work has given the greatest satisfaction to the reviewer. Statistical and authoritative discussion of rare and unusual subjects, and all in a condensed and practical manner, makes the work indispensable to all classes of the profession.

